# Dr. Albert Moll and a Neglected Aspect of Pedagogy

**Published:** 1912-07-06

**Authors:** 


### CHILD PSYCHIATRY.

### Dr. Albert Moll and a Neglected Aspect of Pedagogy.

THE importance of initiating the child into the elementary facts of biology—and more especially into those which concern him more intimately than do the life histories of the lumbricus and the snail, now so generally accepted as fit examples for biological instruction in the lower departments of "advanced" schools—is becoming increasingly recognised. The matter itself is not a new evolution in pedagogy. It was mooted by old authorities, notably by Pestalozzi and Froebel; but it is only in recent years—practically during the last decade of the nineteenth century—that it has been popularised, if one may use such a phrase, in connection with what is still a semi-tabooed subject. In Germany, Russia, and Italy sexual instruction (a bad term admittedly) is now recognised as an orthodoxy. There is still considerable discussion as to the manner in which such instruction should be given. Add to this the subsidiary questions: Who is to give the instruction? to whom it should be given? and, lastly, but most important of all, how far should the teaching extend? and it will be seen that the problem is not without technical difficulties, while the *suppressio veri* evasion is certainly not unattended with dangers.

Dr. Albert Moll, in his book "The Sexual Life of the Child" (which has been translated into excellent English by Dr. Eden Paul, is published by George Allen and Co., of Rathbone Place, and, as the publisher's note tells us, is only to be sold to the professional classes and to teachers at 15*s.* net), has dealt with the whole subject in a lucid and comprehensive manner. Dr. Moll is an authority of European reputation, and, although his views are not always in strict accordance with those held by other authorities, he is, nevertheless, entitled to careful and respectful consideration when he deals with matters which may rightly be styled his specialty. He has been fortunate in his translator, for Dr. Paul is already well known through his admirable translations of the works of Bloch, Lombroso, and Kisch. The book is well worth reading, not only by those to whom, by the limitations of the publishers, it is now accessible, but by every parent who is interested in the welfare of his children. It is true that it contains nothing that is strikingly original to any medical man who is acquainted with the work of Havelock Ellis in this country and of Freud, Eulenberg, Bloch, and Tardieu on the Continent, but it is a serious attempt to epitomise, in what may be called popular language, the salient facts of that work in the departments more closely connected with children.

### ENVIRONMENT AND THE NORMAL CHILD.

Dr. Moll starts by dealing with the normal child, and proceeds to show in what way, and by what conditions of environment, education, and inherited temperament, the normal development of the sexual life of the child may be interfered with. The chapter dealing with pathology—more correctly with psychiatry—is one of the best and clearest expositions of a very difficult subject that we have had the pleasure of reading. While we do not agree with the author on several points, we can honestly congratulate him on the attempt to give a résumé impartial enough to prove illuminative to many teachers and parents. In our own medical schools, unfortunately, no systematic instruction is given in sexual psychiatry. So popular a text-book as Dr. Craig's "Pyschological Medicine" deals scantily with this important phase, and even the larger manuals—we may instance Dr. Mercier's admirable chapters in Allbutt's "System"—attempt to dismiss equally important matters in a few lines of text. The result of this want of teaching is that many medical men are totally ignorant of modern conceptions of sexual psychiatry, especially those which relate to the child. A striking example of such ignorance is found in some letters which recently appeared in a popular semi-medical journal devoted to pediatrics, in which some psychiatric questions were handled in a fashion that would be ludicrous were it not for the fact that there is a disastrous side to such immature treatment. For this reason we commend Dr. Moll's book to all practitioners.

### AN EDUCATIONAL MAXIM.

One of the most important chapters is that devoted to education. The author deals quite frankly with such matters as corporal punishment, the relation between teacher and child, the development of the sexual instinct and the anomalies and perversions that may be produced, not by congenital predisposition but by immoral example and precept. Indeed, with regard to these last, Dr. Moll seems to deny, although he does not implicitly state as much, the possibility of congenital psychiatric states such as those on which Bloch and Forel, and Krafft-Ebing, in his later works, have laid special stress. This method of handling a foolish taboo is due to the fact that the author clearly recognises (page 248) "that in the education of the child the complete exclusion of sexual stimuli is impossible"—a conclusion with which every teacher of experience will agree. Equally evident is the truth of his remark on the opposite page: "A child may receive the best of instruction without result, if in its own environment it is continually seeing something precisely the opposite of that which it is being told. This applies with equal force to the sexual life, which can be influenced far more readily by example than by good teaching, if the latter, though daily repeated, conflicts with what the child sees every day in the conduct of its relatives and companions." Naturally, the first thing therefore that is necessary, if parents and teachers are to influence the sexual life of children with advantage, is that both should be acquainted with the important facts bearing on the sexual life and with the possible results of aberrations and abnormalities.

Like Cervantes, Dr. Moll has not much patience with the ignorant parent or teacher. "A father," he remarks, "who starts with the false assumption that his son must inevitably have intercourse with so many prostitutes and must seduce so many girls —in a word, a father who regards sexual abstinence as unmanly, or as necessarily dangerous to health (and fathers who hold such opinions are no rarity)—such a father must himself be sexually enlightened before we give him the right to enlighten his son." Dr. Moll nowhere thinks himself what a lawyer would term estoppeled from tearing aside the conventional veils that have been allowed to conceal the facts. He cites instances from his case-books proving what harm—not merely moral, but physical and mental—results from trying to evade his first premiss that it is possible to withhold from children all sexual stimuli. It is this outspokenness which makes his book so valuable.

### The Mental Life of the Child.

He is equally sane in dealing with the manner in which sexual instruction should be imparted to the young. He recognises that there are wide differences in what he calls psycho-sexual puberty in children. "For the fulfilment of the aims of the sexual enlightenment," he says, "it does not so much matter when the first physical manifestations of puberal development make their appearance, but when the first sexual feelings and sentiments, which must be distinguished from the unconscious and purely physical symptoms, are experienced . . . The important matter is, not whether follicles have already matured in the ovary, but what influence such a process has exercised upon the mental life of the child. For this reason, in our study of the individual case, we must have some knowledge of the psyche of the child with which we are concerned." This is a wise warning. No good will be gained by systematic biological instruction given to a class of boys and girls of even approximately the same ages. The parent and teacher must discriminate and select most carefully the children to be enlightened. In some cases the teaching will have to be early: in others it may safely be postponed to a later date. The fact remains that only by individualising can we hope to do good. Dr. Moll is in accord with Paget and a host of other authorities in regard to the harmfulness of leaving such instruction till it is too late. The vicious attentions of quacks and the obscenities of advertisements in the press and in public places influence the sexual life of the growing child detrimentally. This is well known to every doctor.

But it is scarcely the doctor who can guard against such influence. The defence must come from the parent or teacher primarily. Only in certain cases is the family medical attendant appealed to, and then it is usually found that there is already a taint which may or may not be curable. We cannot here enter into this subject fully, but Dr. Moll has dealt with it so trenchantly that little need be said to support his views. Whether parents and teachers agree with his opinions or not, they will at all events be certain of finding that he puts forward a case which is not adulterated by sentimentality but which rests on foundations of solid fact, and is strengthened by logical deductions drawn from scientifically reliable evidence.

